# Complete genome sequence of *Kosakonia sacchari* type strain SP1^T^

**DOI:** 10.4056/sigs.5779977

**Published:** 2014-06-06

**Authors:** Mingyue Chen, Bo Zhu, Li Lin, Litao Yang, Yangrui Li, Qianli An

**Affiliations:** 1 State Key Laboratory of Rice Biology, Institute of Biotechnology, Zhejiang University, Hangzhou, China; 2 Guangxi Key Laboratory of Sugarcane Genetic Improvement, Sugarcane Research Institute, Guangxi Academy of Academy of Agricultural Sciences, Nanning, China; 3 State Key Laboratory for Conservation and Utilization of Subtropical Agro-bioresources, Guangxi University, Nanning, China

**Keywords:** endophyte, *Enterobacter*, *Kosakonia*, nitrogen fixation, plant growth-promoting bacteria, sugarcane

## Abstract

*Kosakonia sacchari* sp. nov. is a new species within the new genus Kosakonia, which was included in the genus Enterobacter. *K sacchari* is a nitrogen-fixing bacterium named for its association with sugarcane (*Saccharum officinarum* L.). *K sacchari* bacteria are Gram-negative, aerobic, non-spore-forming, motile rods. Strain SP1^T^ (=CGMCC1.12102^T^=LMG 26783^T^) is the type strain of the *K sacchari* sp. nov and is able to colonize and fix N_2_ in association with sugarcane plants, thus promoting plant growth. Here we summarize the features of strain SP1^T^ and describe its complete genome sequence. The genome contains a single chromosome and no plasmids, 4,902,024 nucleotides with 53.7% GC content, 4,460 protein-coding genes and 105 RNA genes including 22 rRNA genes, 82 tRNA genes, and 1 ncRNA gene.

## Introduction

The genus Enterobacter belonging to the family Enterobacteriaceae is polyphyletic based on 16S rRNA gene sequence analysis [1-3]. Recently, eleven species belonging to the genus Enterobacter were transferred into the genus Cronobacter and three novel genera (Lelliottia, Pluralibacter, and Kosakonia) based on multilocus sequence analysis of protein-coding genes, *rpoB* (RNA polymerase β-subunit gene), *gyrB* (DNA gyrase subunit B gene), *infB* (initiation translation factor 2 gene), and *atpD* (ATP synthase β-subunit gene) [1]. Enterobacter cowanii, E. radicincitans, E. oryzae and E. arachidis were reclassified as Kosakonia cowanii, K. radicincitans, K. oryzae and K. arachidis, respectively [1]. Enterobacter sacchari is a new species named for nitrogen-fixing bacteria in association with sugarcane (*Saccharum officinarum* L.) [2,4] and has been reclassified as *Kosakonia sacchari* [3]. *K sacchari* is able to colonize sugarcane plants, fix N_2_ in association with sugarcane plants and promote plant growth [4]. *K sacchari* strain SP1^T^ was isolated from a surface-sterilized stem of sugarcane cultivar GT11 grown in Nanning, Guangxi, China in 1994. It has now been designated the type strain of *K sacchari* sp. nov [2,3]. Here we present a summary of its features [2] and the complete genome sequence and annotation for *K sacchari* strain SP1^T^ (=CGMCC1.12102^T^=LMG 26783^T^).

## Organism information

### Classification and general features

**Figure 1:**
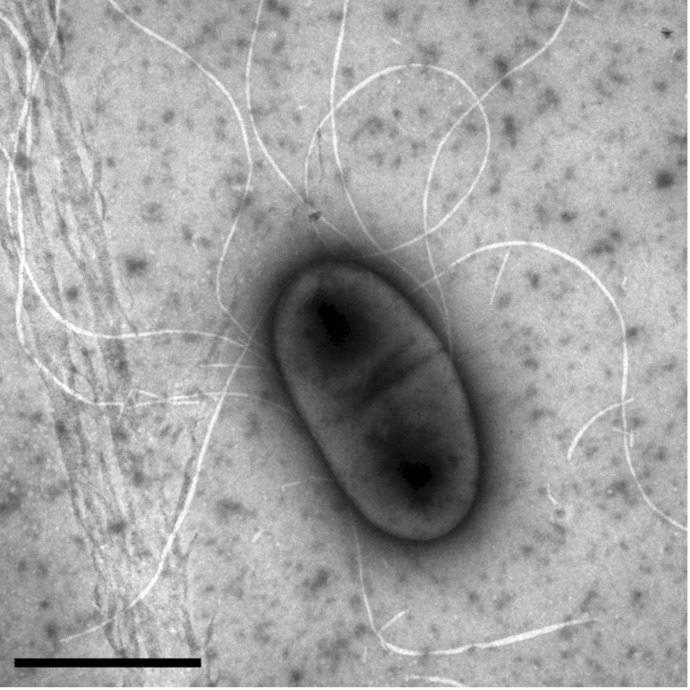
Transmission electron micrograph showing a negative-stained cell of the *Kosakonia sacchari* type strain SP1^T^ [2]. The scale bar represents 1 μm.

*K sacchari* type strain SP1^T^ is a Gram-negative, non-spore-forming, motile rod with peritrichous flagella (Figure 1., Table 1.[2]). It grows aerobically but reduces N_2_ to NH_3_ at a low pO_2_. It is able to grow and fix N_2_ on media containing 10% (w/v) cane sugar or sucrose and forms circular, convex, smooth colonies with entire margins on solid media. It grows best around 30°C and pH 7.

**Table 1:** Classification and general features of *Kosakonia sacchari* type strain SP1^T^ according to the MIGS recommendations

**MIGS ID**	**Property**	**Term**	**Evidence code**
	Current classification	Domain Bacteria Phylum Proteobacteria Class Gammaproteobacteria Order Enterobacteriales Family Enterobacteriaceae Genus Kosakonia Species *Kosakonia sacchari* Type strain: SP1^T^	TAS [16] TAS [17] TAS [18-20] TAS [21] TAS [22,23] TAS [1,3] TAS [2,3] TAS [2,3]
	Gram stain	Negative	TAS [2]
	Cell shape	Rod	TAS [2]
	Motility	Motile	TAS [2]
	Sporulation	Non-sporulating	TAS [2]
	Temperature range	Mesophile	TAS [2]
	Optimum temperature	28 – 32°C	TAS [2]
	Carbon source	Sucrose, glucose, fructose, galactose, maltose, mannitol, mannose, arabitol	TAS [2]
	Energy source	Chemoorganotroph	TAS [2]
MIGS-6	Habitat	Soil, plants	IDA
MIGS-6.3	Salinity	0 – 4% NaCl	TAS [2]
MIGS-22 MIGS-23	Oxygen Isolation	Aerobe Stem of sugarcane cultivar GT11	TAS [2] TAS [2]
MIGS-15	Biotic relationship	Free-living, endophytic	IDA
MIGS-14	Pathogenicity	Not reported	
MIGS-4	Geographic location	Nanning, Guangxi, China	TAS [2]
MIGS-5	Sample collection time	1994	TAS [2]
MIGS-4.1 MIGS-4.2	Longitude Latitude	108.33 22.84	NAS NAS
MIGS-4.3	Depth	0.1 – 0.5 m above the surface	IDA
MIGS-4.4	Altitude	76 m	NAS

Evidence codes - IDA: Inferred from Direct Assay; TAS: Traceable Author Statement (i.e., a direct report exists in the literature); NAS: Non-traceable Author Statement (i.e., not directly observed for the living, isolated sample, but based on a generally accepted property for the species, or anecdotal evidence).

**Figure 2:**
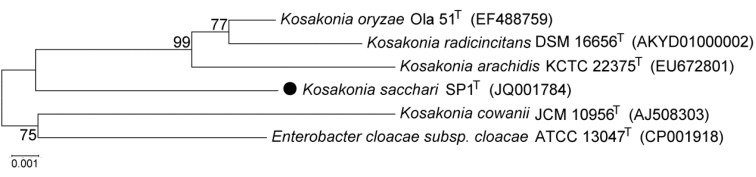
Phylogenetic tree based on 16S rRNA gene sequences of *Kosakonia sacchari* type strain SP1^T^ (●), the type strains of other species in the genus Kosakonia, and the type strain of Enterobacter cloacae. The sequences were aligned with the CLUSTAL W program and were constructed with the neighbor-joining algorithm integrated in the MEGA 5.0 program [5]. The phylogenetic tree was tested with 1,000 bootstrap replicates. Bootstrap values are shown at the nodes. The GenBank accession numbers of the sequences are indicated in parentheses. The scale bar represents a 0.1% nucleotide sequence divergence.

Phylogenetic analysis of the 16S rRNA gene sequences from SP1^T^, the type strains of species of the genus Enterobacter and the type strains of type species of other genera in the family Enterobacteriaceae showed that SP1^T^ formed a monophyletic group with the type strain of E. cloacae (the type species of the genus Enterobacter) [2]. However, phylogenetic analysis of the *rpoB* gene sequences showed that SP1^T^ diverged from E. cloacae [2]. Here, phylogenetic analysis of the 16S rRNA gene sequences from SP1^T^, other type strains in the genus Kosakonia, and the type strain of E. cloacae showed that *K sacchari* formed a monophyletic group with K. radicincitans, K. oryzae, and K. arachidis and diverged from K. cowanii (the type species of the genus Kosakonia) and E. cloacae (Figure 2.).

Like typical members in the genera Enterobacter and Kosakonia, *K sacchari* SP1^T^ utilizes L-alanine, D-cellobiose, citrate, D-fructose, D-galactose, D-glucose, glycerol, maltose, D-mannitol and D-mannose [2,6,7]. *K sacchari* differentiates from E. cloacae by utilization of D-arabitol and L-fucose, differentiates from K. radicincitans by utilization of putrescine, D-arabitol, L-fucose and *α*-methyl-D-glucoside, and differentiates from K. oryzae by utilization of putrescine, D-arabitol and L-rhamnose [2].

## Genome sequencing information

### Genome project history

*K sacchari* SP1^T^ was selected for sequencing because it is the type strain of *K sacchari*, and on the basis of its scientific interest as an endophyte that has the potential to promote the growth of agriculturally important crops by nitrogen fixation [8]. Its 16S rRNA gene sequence is deposited in GenBank under the accession number JQ001784. Its genome sequence is deposited in GenBank under the accession number CP007215.2. A summary of the genome sequencing project information and its association with MIGS version 2.0 compliance is shown in Table 2.

**Table 2:** Genome sequencing project information for *Kosakonia sacchari* type strain SP1^T^

**MIGS ID**	**Property**	**Term**
MIGS-31	Finishing quality	Finished
MIGS-28	Libraries used	Pacbio 4 – 10 Kb library
MIGS-29	Sequencing platforms	PacBio RS II
MIGS-31.2	Fold coverage	63 ×
MIGS-30	Assemblers	HGAP in smrtanalysis-2.1.1
MIGS-32	Gene calling method	GeneMarkS+
	Genome Database release	Genbank
	Genbank ID	CP007215.2
	Genbank Date of Release	May 23, 2014
	Project relevance	Taxonomy, biotechnology

### Growth conditions and DNA isolation

*K sacchari* SP1^T^ was grown in liquid Luria-Bertani (LB) medium at 30°C to early stationary phase. The genome DNA was extracted from the cells by using a TIANamp bacterial DNA kit (Tiangen Biotech, Beijing, China). DNA quality and quantity were determined with a Nanodrop spectrometer (Thermo Scientific, Wilmington, USA).

### Genome sequencing and assembly

The genome DNA of *K sacchari* strain SP1^T^ was first constructed into a 500-bp-insert library and sequenced by an Illumina HiSeq 2000 sequencing system. A draft genome of 4,945,084 nucleotides containing 239 contigs was obtained and deposited at DDBJ/EMBL/GenBank under the accession no. AMSC00000000 [8]. However, 84,628 nucleotides (203 short contigs) of the draft genome were accidently contaminated by sequences from eukaryotic organisms. Therefore, the genome of SP1^T^ was resequenced at the Duke University Genome Sequencing & Analysis Core Resource using the Pacific Biosciences’ Single Molecule, Real-Time (SMRT) sequencing technology (http://www.pacificbiosciences.com/). A 4 – 10 Kb insert library was constructed. Sequencing was run on a single SMRT Cell. The sequencing data were assembled using the Hierarchical Genome Assembly Process (HGAP) with smrtanalysis-2.1.1. The final assembly of the chromosome produced 63-fold coverage of the genome.

### Genome annotation

Automated genome annotation was completed using the NCBI Prokaryotic Genome Annotation Pipeline. Product description annotations were obtained using searches against the KEGG, InterPro, and COG databases. Genes with signal peptides were predicted using SignalP [9]. Genes with transmembrane helices were predicted using TMHMM [10]. Genes for tRNA were found by tRNAScanSE [11]. Ribosomal RNAs were found by using BLASTN vs. ribosomal RNA databases, and 5S rRNA hits were further refined using Cmsearch (http://manpages.ubuntu.com/manpages/raring/man1/cmsearch.1.html). Two hundred twenty seven disrupted genes were replaced by the complete gene sequences obtained from the first Illumina HiSeq 2000 sequencing.

## Genome properties

**Figure 3:**
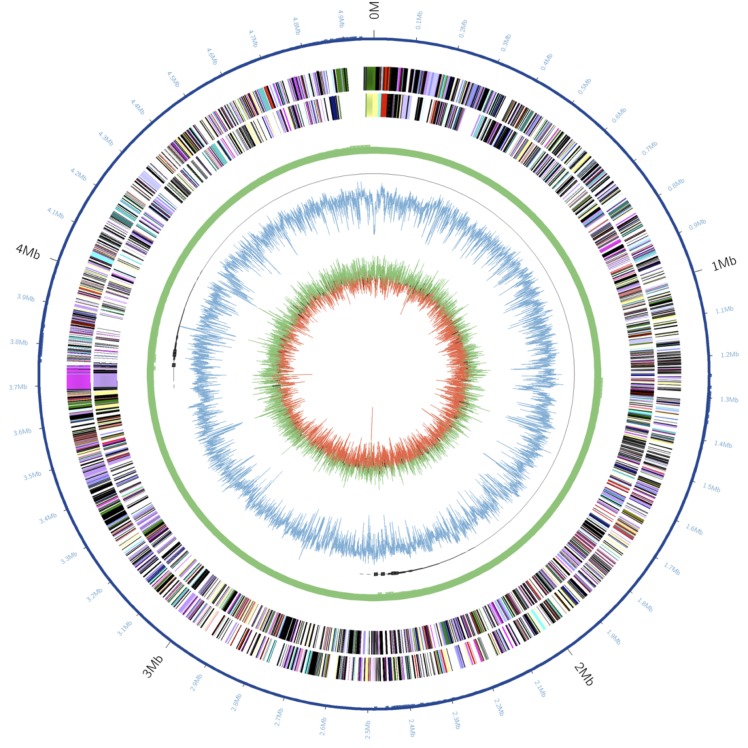
Graphical circular map of the chromosome of *Kosakonia sacchari* type strain SP1^T^. From outside to the center: Genes on forward strand (color by SEED subsystems [12]), Genes on reverse strand (color by SEED subsystems), genome structure (a circular chromosome with no gaps), GC content, GC skew.

The genome of *K sacchari* SP1^T^ contains a single chromosome of 4,902,024 nucleotides with 53.7% GC content and no plasmids (Table 3, Figure 3.). The genome contains 4,585 predicted genes, 4,460 protein-coding genes and 105 RNA genes including 22 rRNA genes, 82 tRNA genes and 1 ncRNA gene. A total of 3,752 genes (81.8%) were assigned a putative function. The remaining genes were annotated as hypothetical or unknown proteins (Table 3). The distribution of genes into COGs functional categories is presented in Table 4.

**Table 3:** Nucleotide content and gene count levels of the genome

Attribute	Value	% of total
Size (bp)	4,902,024	100.00
G+C content (bp)	2,634,551	53.74
Coding region (bp)	4,281,189	87.34
Total genes	4,585	100.00
RNA genes	105	2.29
Protein-coding genes	4,460	97.27
Pseudo genes	20	0.44
Genes assigned to COGs	3,786	82.57
Genes with signal peptides	452	9.86
Genes with transmembrane helices	1096	23.90

**Table 4:** Number of genes associated with the 25 general COG functional categories

**Code**	**Value**	**% of total**^a^	**Description**
J	193	4.33	Translation
A	2	0.04	RNA processing and modification
K	386	8.65	Transcription
L	170	3.81	Replication, recombination and repair
B	0	0.00	Chromatin structure and dynamics
D	38	0.85	Cell cycle control, mitosis and meiosis
Y	0	0.00	Nuclear structure
V	52	1.17	Defense mechanisms
T	269	6.03	Signal transduction mechanisms
M	251	5.63	Cell wall/membrane biogenesis
N	128	2.87	Cell motility
Z	0	0.00	Cytoskeleton
W	0	0.00	Extracellular structures
U	107	2.40	Intracellular trafficking and secretion
O	144	3.23	Posttranslational modification, protein turnover, chaperones
C	268	6.01	Energy production and conversion
G	394	8.83	Carbohydrate transport and metabolism
E	414	9.28	Amino acid transport and metabolism
F	90	2.02	Nucleotide transport and metabolism
H	186	4.17	Coenzyme transport and metabolism
I	117	2.62	Lipid transport and metabolism
P	265	5.94	Inorganic ion transport and metabolism
Q	82	1.84	Secondary metabolites biosynthesis, transport and catabolism
R	481	10.78	General function prediction only
S	382	8.57	Function unknown
-	674	15.11	Not in COGs

a) The total is based on the total number of protein coding genes in the annotated genome.

## Comparison with the genome of Enterobacter sp. strain R4-368

The chromosome of *K sacchari* SP1^T^ shows the highest sequence similarities ranging from 69.5% to 100% to the chromosome of Enterobacter sp. strain R4-368, which is an endophytic nitrogen-fixing bacterium isolated from the biofuel plant *Jatropha curcas* [13]. The genome of the strain R4-368 comprises a single circular chromosome of 5,039,027 bp with 54.0% GC content (deposited in GenBank under the accession number CP005991) and one plasmid pENT01 of 116,007 bp with 52.8% GC content (deposited in GenBank under the accession number CP005992) [13].

The chromosome of *K sacchari* SP1^T^ shares 4,105 genes (89.5%) with the chromosome of strain R4-368. The digital DNA-DNA hybridization values between the two chromosomes calculated by the online Genome-to-Genome Distance Calculator [14,15] (version 2.0; http://ggdc.dsmz.de) are 90.2%, 57.7%, and 86.6% under the distance Formula 1, 2 (recommended for dealing with incomplete genomes), and 3, respectively. The probabilities of same species for the two strains (DDH > 70%) assessed via logistic regression are 97.4%, 44.3%, and 98.8%, respectively. Likely, strain R4-368 belongs to the species *K sacchari*.
